# Pompe disease: from pathophysiology to therapy and back again

**DOI:** 10.3389/fnagi.2014.00177

**Published:** 2014-07-23

**Authors:** Jeong-A Lim, Lishu Li, Nina Raben

**Affiliations:** Laboratory of Muscle Stem Cells and Gene Regulation, National Institute of Arthritis and Musculoskeletal and Skin Diseases (NIAMS), National Institutes of HealthBethesda, MD, USA

**Keywords:** autophagy, lysosome, Pompe disease, lipofuscin, enzyme replacement therapy

## Abstract

Pompe disease is a lysosomal storage disorder in which acid alpha-glucosidase (GAA) is deficient or absent. Deficiency of this lysosomal enzyme results in progressive expansion of glycogen-filled lysosomes in multiple tissues, with cardiac and skeletal muscle being the most severely affected. The clinical spectrum ranges from fatal hypertrophic cardiomyopathy and skeletal muscle myopathy in infants to relatively attenuated forms, which manifest as a progressive myopathy without cardiac involvement. The currently available enzyme replacement therapy (ERT) proved to be successful in reversing cardiac but not skeletal muscle abnormalities. Although the overall understanding of the disease has progressed, the pathophysiology of muscle damage remains poorly understood. Lysosomal enlargement/rupture has long been considered a mechanism of relentless muscle damage in Pompe disease. In past years, it became clear that this simple view of the pathology is inadequate; the pathological cascade involves dysfunctional autophagy, a major lysosome-dependent intracellular degradative pathway. The autophagic process in Pompe skeletal muscle is affected at the termination stage—impaired autophagosomal-lysosomal fusion. Yet another abnormality in the diseased muscle is the accelerated production of large, unrelated to ageing, lipofuscin deposits—a marker of cellular oxidative damage and a sign of mitochondrial dysfunction. The massive autophagic buildup and lipofuscin inclusions appear to cause a greater effect on muscle architecture than the enlarged lysosomes outside the autophagic regions. Furthermore, the dysfunctional autophagy affects the trafficking of the replacement enzyme and interferes with its delivery to the lysosomes. Several new therapeutic approaches have been tested in Pompe mouse models: substrate reduction therapy, lysosomal exocytosis following the overexpression of transcription factor EB and a closely related but distinct factor E3, and genetic manipulation of autophagy.

## Background and history

Pompe disease, also known as glycogen storage disease type II (GSDII) or “acid maltase deficiency”, is caused by the absence or deficiency of acid alpha-glucosidase (GAA), a lysosomal enzyme that is responsible for the cleavage of the α-1,4- and α-1,6-glycosidic bonds of glycogen to glucose. The deficiency of the enzyme leads to the accumulation of glycogen in the lysosomes in numerous tissues, but clinical symptoms are primarily due to cardiac and skeletal muscles involvement. The disease is characterized by a wide variety of manifestations ranging from severe infantile-onset muscle weakness, hypotonia, and hypertrophic cardiomyopathy to a relatively mild slowly progressive skeletal muscle myopathy in adults (Hirschhorn and Reuser, 2001). Pompe disease is a rare disorder with an estimated frequency of 1:40,000 (Martiniuk et al., 1998; Ausems et al., 1999). The neonatal screening program for lysosomal storage diseases (LSDs) in Taiwan and Austria using dried blood spots revealed a higher than expected frequency of the disease in these populations (Chien et al., 2008; Mechtler et al., 2012).

In 1932, Johannes Cassianus Pompe, a Dutch pathologist, described the disease in a 7-month-old infant who died of idiopathic hypertrophy of the heart; in addition to the cardiac problems, the infant had generalized muscle weakness. Dr. Pompe made the crucial observation that the baby’s symptoms were associated with massive “vacuolar” glycogen storage in virtually all tissues; reports of similar cases appeared in literature the same year (Bischoff, 1932; Putschar, 1932) as well as in the following years. In 1954 the disease was classified as glycogen storage disease type II to reflect the abnormal metabolism of glycogen (Cori, 1954). However, at that time, the cause of the disease, the “vacuolar” nature of the storage, and the apparent normal molecular structure of the accumulated glycogen all remained a mystery. The connection between lysosomes, the enzyme defect, and Pompe disease was made much later, in 1963, by a Belgian biochemist Henri-Gery Hers. He discovered a new enzyme (maltase) that carried out the hydrolysis of glycogen to glucose at an acidic pH, and he demonstrated that the enzyme was absent in patients with Pompe disease (Hers, 1963). By a fortunate coincidence, Dr. Hers had been previously working in the laboratory of Christian de Duve, who in 1955 postulated the existence of a new group of intracellular organelles, lysosomes. Dr. Hers realized that his new enzyme resides in the lysosomes, and that this is the only glycogen-degrading enzyme present in the lysosomes. Based on this research, the concept of LSDs has been established, and the search for other missing lysosomal enzymes began. Pompe disease became the first in a diverse group of more than 50 currently recognized lysosomal storage disorders. The cause of Pompe disease became known 30 years after its initial description, and it would take even longer to develop the first therapy.

## Genetic defects and clinical manifestations

Pompe disease comes at different ages and with different degrees of severity. The severity of the clinical presentations, the tissue involvement, and the age of onset generally correlate well with the nature of the mutation and the degree of residual enzyme activity. A database containing all the reported mutations and polymorphisms of the *GAA* gene on chromosome 17 q25 (Hoefsloot et al., 1988, 1990; Martiniuk et al., 1990; Kuo et al., 1996) (>300 variants) may be accessed at http://www.pompecenter.nl. The majority of mutations are private—found only in a single family or a small population—and most patients are compound heterozygotes. The mutations are spread throughout the gene and affect one of the multiple steps involved in synthesis, posttranslational modifications, lysosomal trafficking, and proteolytic processing of GAA (see below).

The most common mutation among Caucasian children and adults is c.-32-13T>G (IVS1), a splicing defect which allows for the synthesis of low levels (~10–20%) of normal enzyme (Huie et al., 1994; Boerkoel et al., 1995; Raben et al., 1996). A study of a large cohort of patients with a similar genotype—IVS1 in combination with a second null mutation—unexpectedly showed a significant variability in the age at disease onset, suggesting the role of secondary modifying factors on the clinical course (Kroos et al., 2012). One of the possible factors responsible for the wide clinical variability has recently come to light: the deletion/deletion polymorphism in the gene coding for the angiotensin-converting enzyme (ACE), which is known to increase the number of type II fibers and influence muscle properties, is associated with earlier onset, higher creatine kinase (CK) levels, muscle pain, and more severe progression of the disease in patients with adult form (de Filippi et al., 2010).

Two mutations—c.del525T and exon18 deletion—are frequent in the Netherlands (Hermans et al., 1994; Hirschhorn and Huie, 1999), and a common defect, c.1935C>A (p.Asp645Glu), is shared by Chinese patients in Taiwan (Shieh and Lin, 1998). The most common mutation in African-Americans, c.2560C>T (p.Arg854Ter), most likely originated in their ancestral population from north-central Africa and was brought to the Americas during the slave trade (Becker et al., 1998).

The most severe end of the phenotypic continuum is the disease in which symptoms begin within the first months of life, and include profound muscle weakness, hypotonia (“floppy baby”), and hypertrophic cardiomyopathy. Massive cardiomegaly, which is easily detectable by chest X-rays, is one of the leading manifestations of the disease in infants. The weakness of respiratory muscle and cardiomegaly often lead to diminished ventilation and frequent infections. Macroglossia, mild hepatomegaly, feeding difficulties, and significantly delayed motor milestones are also typical manifestations of this rapidly progressive form; most patients do not survive beyond the first year of life and die from cardiac failure. This severe subtype, described by Dr. Pompe, is called the classic infantile form, and is caused by complete or near complete loss of GAA activity (van den Hout et al., 2003; Kishnani et al., 2006). Similar clinical presentations in infants with less severe cardiomyopathy, absence of left ventricular outflow obstruction, and somewhat longer survival have been classified as a non-classical infantile form (Slonim et al., 2000).

Partial loss of enzyme activity (residual enzyme activity between 1 and 30%) manifests as progressive muscle dysfunction and respiratory insufficiency without cardiac involvement. A spectrum of phenotypic variation and genotypic heterogeneity is characteristic of this less dismal form of the disease. The lower limbs and paraspinal muscles are frequently affected first, followed by the respiratory muscle, particularly the diaphragm, intercostal, and accessory muscles. As the disease progresses, patients may develop severe scoliosis and lumbar hyperlordosis and many become wheelchair dependent and require assisted ventilation. Respiratory failure is the main cause of increased morbidity and mortality (Herzog et al., 2012; Schuller et al., 2012). Although skeletal muscle weakness dominates the clinical picture, there is increasing evidence of the involvement of non-muscle tissues in this group of patients (Filosto et al., 2013).

A variety of scientific terms are used in literature to describe these different forms of the disease, such as typical and atypical infantile, non-infantile, muscular, childhood-, juvenile-, and adult-onset, etc.; the broad term, late-onset Pompe disease (LOPD), is often used to describe patients with muscle weakness without cardiac involvement and the onset of symptoms after 12 months of age. However, the continuum of phenotypes often defies categorization. Recently, the Pompe community accepted the following proposed nomenclature: (1) “classic infantile”, as described above; (2) “childhood” form with onset of symptoms from birth till adolescence without persisting and progressive cardiac hypertrophy; and (3) “adult” form with onset of symptoms from adolescence to late adulthood (Güngör and Reuser, 2013).

## Acid alpha-glucosidase and Enzyme Replacement Therapy (ERT)

Like many other lysosomal enzymes, GAA is synthesized in the rough endoplasmic reticulum (ER), where high mannose oligosaccharides are added to the 110 kDa precursor molecules, a process known as glycosylation (Hermans et al., 1993). The glycosylation and proper folding in the ER are essential for the trafficking of the enzyme through the Golgi complex to the lysosomes. On the way to the lysosomes, the protein undergoes sugar chain modifications and proteolytic cleavage (Wisselaar et al., 1993). In the Golgi, the addition of the mannose-6-phosphate (M6P) moiety (phosphorylation) allows for the recognition of the enzyme by (M6P) receptors, which transport the enzyme to early and late endosomes. The Golgi is also a site for the first proteolytic cleavage of the precursor followed by the additional cleavage at both the amino- and carboxyl-terminal ends before and after entry to the lysosomes (Wisselaar et al., 1993; Moreland et al., 2005). The posttranslational modifications of the precursor protein, a process called maturation, increase its activity of the enzyme for glycogen (Wisselaar et al., 1993). GAA along with other lysosomal enzymes leaves the Golgi complex in a vesicle which delivers its content to early/late endosomes and lysosomes. Once inside the late endosomes, the receptor-ligand complexes dissociate due to the low pH in these vesicles, and the enzyme is delivered to the lysosome, whereas the receptors recycle back for the next round of sorting (Kornfeld, 1992). A portion of the GAA precursor is secreted, and can be taken up by neighboring cells via cation independent mannose 6-phosphate receptor (CI-MPR) on the plasma membrane, which directs the endocytosis and transport of the enzyme to the lysosome. The ability of cells to secrete and internalize lysosomal enzymes, first demonstrated in cross-correction experiments, in which normal cells rescued the nearby deficient cells (Neufeld and Fratantoni, 1970), became the fundamental basis of enzyme replacement therapy (ERT) for lysosomal storage diseases, including Pompe disease.

In Pompe disease, the CHO-produced recombinant human GAA (rhGAA; alglucosidase alpha, Myozyme®, Genzyme Corporation, Framingham, MA) is a 110 kDa precursor containing M6P groups that enable the enzyme to bind the receptor on the cell surface. Once inside the cell, the rhGAA, like the endogenous precursor, is cleaved to yield intermediate and fully mature 76 and 70 kDa lysosomal forms. In 2006 the drug received broad-label marketing approval in Europe, and later in the U.S. This is the first therapy for GSDII, and the first attempt to direct recombinant enzyme to skeletal muscle. This therapy is based on a straightforward hypothesis to explain the disease pathogenesis, namely that the progressive enlargement of glycogen-filled lysosomes, lysosomal rupture (due to mechanical pressure in muscle fibers), and release of glycogen and toxic substances into the cytosol would ultimately result in organ dysfunctions (Griffin, 1984a; Thurberg et al., 2006). The stages and progression of skeletal muscle damage have been described for the classical infantile form: small glycogen-filled lysosomes in between intact myofibrils are typical for stage 1; an increase in cytoplasmic glycogen and the size and number of lysosomes combined with fragmentation of myofibrils constitute stage 2; after that, glycogen-filled lysosomes are tightly packed, some show membrane rupture, and only few myofibril fragments remain in stage 3; finally, in stages 4 and 5, most glycogen is cytoplasmic, the contractile elements of muscle cells are completely lost, and the cells bloat due to the influx of water (Thurberg et al., 2006).

The assumption was that early treatment, initiated before lysosomal integrity was compromised, would reverse this pathogenic cascade and cure the disease. The outcome, however, was somewhat unexpected—cardiac muscle responded remarkably well to therapy, but skeletal muscle did not. Even with extremely high dosages of the drug (20–40 mg/kg body weight, which is significantly higher than in other LSDs), patients with the childhood and adult forms of the disease experience limited clinical benefit, such as modest improvements in walking distance and respiratory function, but skeletal muscle weakness often persists, and some show signs of disease progression (Van den Hout et al., 2004; Kishnani et al., 2007; Schoser et al., 2008; Strothotte et al., 2010; Van der Ploeg et al., 2010; Angelini and Semplicini, 2012).

The reversal of cardiac abnormalities dramatically changed the natural course of the disease in infants; most survive significantly longer compared with untreated group analyzed by retrospective studies (Kishnani et al., 2007; Nicolino et al., 2009). However, the great success of ERT in Pompe disease comes with unintended consequences: many long-term survivors suffer from debilitating skeletal muscle myopathy and develop new previously unrecognized symptoms such as ptosis, hypernasal speech, osteopenia, hearing loss and gastroesophageal reflux. Although the response to therapy varies significantly in this group of patients, an emerging pattern—initial improvement followed by a decline and chronic disability—indicates that even at a very high doses, the drug does not halt the progression of the disease (Chakrapani et al., 2010; Prater et al., 2012, 2013).

One thing appears to be clear: treatment of the infants should start within days after birth, not months (Chien et al., 2009, 2013; Prater et al., 2013). The effect of therapy in pre-symptomatic infants who were diagnosed through a newborn screening program (in Taiwan) was better than in those who were diagnosed later based on clinical symptoms (Chien et al., 2009). It is also clear that sustained high antibody titers on ERT, particularly in infants with cross-reactive immunological material (CRIM)-negative status, are associated with poor outcome (Banugaria et al., 2011).

The neonatal screening program, which allows for the early diagnosis and timely initiation of therapy in infants, would also identify patients with mutations that are associated with onset of symptoms in the second, fourth or six decade of life. The appropriate age for the initiation of therapy in patients with milder forms of the disease is not clear, thus adding to the complexity of ethical issues related to such a program. The argument in favor of such a program for Pompe disease comes from our own experience (however limited), which indicates that the best morphological results are achieved when the therapy is initiated in asymptomatic patients. A case in point is a normal muscle biopsy after only 6 months of therapy, in an infant who was diagnosed through the newborn screening program, but whose genetic makeup was consistent with the childhood form of the disease; the baseline biopsy of this asymptomatic patient with slightly elevated CK level showed well-preserved fibers with minimally enlarged lysosomes and no autophagic buildup (Raben et al., 2010a).

The limitations of current therapy stimulated research on more effective second-generation drugs. The evolving new therapies are designed to improve the delivery of the recombinant enzyme to skeletal muscle. The poor capacity of the current drug to reach skeletal muscle (most of the administered enzyme is taken by liver) has been attributed to the low number of M6P receptors on the plasma membrane of skeletal muscle cells. Therefore, the development of chemically modified recombinant human GAA with high affinity for the receptor and enhanced targeting properties is currently being pursued by academic institutions and pharmaceutical companies (Zhu et al., 2009; Tiels et al., 2012; Maga et al., 2013). Albuterol, a drug that enhances the M6P receptor expression, is also in clinical trial in combination with ERT (Koeberl et al., 2014). Another therapeutic approach includes the use of pharmacological chaperones to increase the stability and half-life of the current drug (Porto et al., 2009, 2012). It is, however, unclear how much enzyme is needed to reverse the established pathology in Pompe skeletal muscle, and this uncertainty remains a concern with these new approaches. It is widely believed that the levels of ~30% of average normal activity would be sufficient, since the disease manifests when the acid α-glucosidase activity drops below this critical threshold. However, our data in transgenic mice expressing human GAA in skeletal muscle of the GAA knockout mice (see Models of Pompe disease) suggest that much higher levels might be needed for the reversal of the advanced disease (Raben et al., 2005). The benefits of these new strategies for enzyme delivery are beyond the scope of this review.

## Gene therapy

A major development in the field is the commencement of the first phase I/II clinical trial of rAAV1-hGAA intramuscular gene transfer. Five children with chronic ventilator dependence (full-time mechanical ventilation despite ERT) and severe phrenic neuromusclular dysfunction were enrolled in the trial. All patients were on ERT, which was continued throughout the study. The outcome of the 180-day safety and ventilatory outcomes following intradiaphragmatic delivery of AAV-mediated *GAA* gene therapy has been reported (Smith et al., 2013). The results indicated that rAAV1-hGAA was safe and led to a modest improvement in ventilatory function. This trial is the first critical step in the development of a successful AAV-based gene therapy for Pompe disease. Extensive preclinical studies (both *in vitro* and *in vivo* in the mouse model) by a number of groups established the basis and feasibility of gene therapy for this disorder; the reader is referred to a review on this subject (Byrne et al., 2011). Here, we will focus on the pathogenesis of skeletal muscle damage in Pompe disease, which turned out to be more complex than previously thought, and involves a profound disturbance of autophagy.

## Models of Pompe disease

Naturally occurring animal models of Pompe disease include cattle, dogs, cats, sheep, and Japanese quails. The underlying genetic defects have been identified in Brahman and Shorthorn cattle breeds in Australia, in quails (AMD quails), and Finnish and Swedish Lapphunds. Curiously, the two Scandinavian dog breeds, which share a common origin and physical appearance, have been recently shown (Seppala et al., 2013) to contain a frameshift mutation similar to that found in patients with the infantile form of the disease (the finding points to a “hot spot” in the *GAA* gene). The presence of residual GAA activity in quails (due to the expression of other alpha-glucosidases) accounts for the milder form of the disease, which mimics human childhood or adult forms without cardiac dysfunction; the birds show progressive muscle weakness, difficulty in lifting their wings or turning from the supine position. With the development of the knockout mouse models, the usefulness of this model became outdated, but AMD quails deserve a special mention because they were the first to be used for testing ERT (Kikuchi et al., 1998).

Genotypically and phenotypically more accurate models were made in mice by targeted disruption of exon 6 and exon 13 of the *GAA* gene [GAA-KO (Raben et al., 1998) and AGLU−/− (Bijvoet et al., 1998)]. These two models have features of both infantile and adult forms: the animals develop cardiomegaly, cardiomyopathy (Bijvoet et al., 1998) and skeletal muscle myopathy, but obvious clinical signs of the disease, such as kyphosis and muscle wasting manifest late relative to their lifespan—at ~7–9 months. Both strains are widely used for testing different therapeutic approaches. Several transgenic lines and double knockouts on the GAA-KO background are available: GFP- LC3:GAA-KO, in which autophagosomes are labeled with green fluorescent protein; GAA-KO:SCID, which do not produce antibody following administration of recombinant human GAA (Xu et al., 2004); and muscle-specific autophagy-deficient GAA-KO, which will be discussed below.

**Figure 1:**
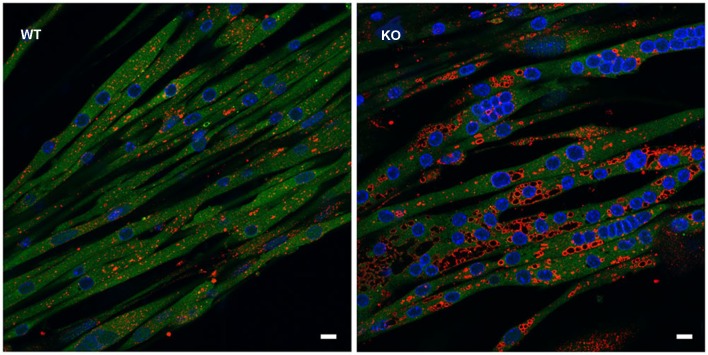
***In vitro* model of Pompe disease replicates lysosomal, but not autophagic pathology**. The images show wild type (**WT**) and GAA-KO (**KO**) myotubes. Immortalized myoblasts were used for the differentiation into multinucleated myotubes. WT myoblasts were derived from mice generated by crosses of GFP-LC3:WT to Immortomice; GAA-KO myoblasts were derived from mice generated by crosses of GFP-LC3:GAA-KO to Immortomice. LC3 (green) is a specific autophagosomal marker. No autophagosomal accumulation is seen in the GAA-KO cells. Myotubes were fixed and stained for lysosomal marker LAMP1 (red). Enlarged lysosomes are seen in the GAA-KO, but not in control WT muscle cells. Bars: 10 μm.

In addition, GAA-KO crosses to H-2K^b^-tsA58 transgenic mice (also called Immortomouse; Charles River Laboratories) allowed for the generation of an *in vitro* model of Pompe disease—GAA-deficient immortalized mouse muscle cell lines. The transgene contains the temperature-sensitive immortalizing SV40 large T antigen tsA58 (tsA58 TAg) under the control of the interferon-inducible H-2K^b^ promoter; the SV40 large T antigen is functional at permissive temperature (33°C plus interferon-γ), but is inactivated at 37° in the absence of interferon. The advantage of this system is that myoblasts derived from the GAA-KO: H-2K^b^-tsA58 mice proliferate and undergo immortalization when the oncogene is expressed, but the differentiation of the cells to myotubes proceeds under “normal” condition when the oncogene is silenced (Jat et al., 1991). Unlike primary myoblasts with their limited proliferation capacity, the immortalized cells can undergo multiple passages without losing the ability to differentiate. The *in vitro* model replicates lysosomal, but not autophagic pathology (see below) (Figure 1; Spampanato et al., 2013), and as such can be used to decipher the early events which precede the development of autophagic buildup—a hallmark of the disease in Pompe muscle fibers.

## Pathogenesis

### Autophagy

At least three autophagic pathways have been described based on the route by which the cargo enters the lysosomes. Microautophagy is a direct engulfment of cytoplasmic components into the lysosomal lumen; in chaperone-mediated autophagy (CMA), a subset of soluble cytosolic proteins with a particular pentapeptide motif are recognized by molecular chaperones and directly translocated into lysosomes through a receptor (LAMP-2A) on the lysosomal membrane; the major third form, most relevant to Pompe disease, is macroautophagy, which fulfils the role of supplying amino acids and energy under starvation by “self-digestion” of intracellular components (Klionsky, 2007; He and Klionsky, 2009; Yang and Klionsky, 2010; Kaushik et al., 2011). Macroautophagy also operates at a low level under a nutrient-rich environment to rid the cells of misfolded proteins, protein aggregates, and worn-out organelles such as mitochondria. Upon induction, the process of macroautophagy (often referred to simply as autophagy) begins with the development of a double membrane, which engulfs part of the cytoplasm, resulting in the formation of the double-membrane structure called autophagosome. Autophagosomes fuses with the lysosome, where the inner membrane and the content of the autophagosome are degraded and recycled. The two pathways, autophagic and endocytic, by which the recombinant human GAA traffics to the lysosomes are interconnected; autophagosomes can fuse with early/late endosomes before fusion with the lysosome, giving rise to an intermediate structure, called amphisome (Berg et al., 1998).

### Autophagy in Pompe mouse models

The morphological evidence for abnormal autophagy in muscle biopsies from adult Pompe patients was first reported by Andrew Engel in 1970 (Engel, 1970), at the time when the field of autophagy was still in its infancy. Perhaps not surprisingly, this pathology and its contribution to the pathogenesis of Pompe disease have been largely ignored until recently. Studies in a GAA-KO model developed in our lab (Raben et al., 1998) clearly demonstrated that abnormalities in Pompe muscle cells were not limited to the enlargement of lysosomes. Analysis of myoblasts derived from GAA-KO mice showed an acidification defect in a subset of late endosomes/lysosomes, a dramatic expansion of all vesicles of the endocytic/autophagic pathways, and a slowdown in the vesicular trafficking in the overcrowded cells (Fukuda et al., 2006).

**Figure 2:**
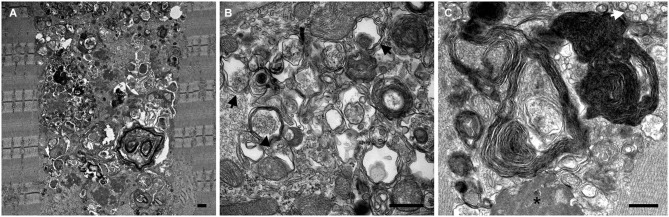
**Electron microscopy images show the presence and the extent (A) of autophagic buildup in skeletal muscle from a 5 month-old GAA-KO mouse**. Larger magnification images **(B and C)** show classical double membrane autophagosomes with undigested cytosolic material (black arrows) or glycogen particles (arrowhead), multimembrane structures (most prominent in **C**), multivesicular body (white arrow), electron dense material (asterisk), as well other cellular debris of unknown origin. Bars: 0.5 μm.

Electron microscopy (EM) of skeletal muscle from the KO mice revealed large areas of autophagic accumulation containing vesicular structures at different stages of a stalled autophagic process: small and large double-membrane autophagosomes with undigested cytosolic material or glycogen particles, multivesicular bodies, multimembrane structures, autofluorescent material, as well other cellular debris (Figure 2). EM established the presence of autophagic accumulation of Pompe muscle, but the extent of this pathology became clear when single muscle fibers were immunostained for lysosomal marker, LAMP1, and autophagosomal marker, LC3, followed by confocal microscopy—an approach best-suited for the detection of autophagic accumulation within the fibers (Raben et al., 2009). Two forms of LC3 are documented: the soluble cytosolic LC3I and lipidated LC3II; the latter remains on the autophagosomal membrane during autophagic process and serves as a highly specific marker of autophagosomes (Kabeya et al., 2000). LAMP1/LC3-double staining showed that the core of the fibers was filled with clusters of densely packed late endosomes/lysosomes and autophagosomes, collectively called autophagic buildup. This buildup often spans the entire length of the fibers, and in many fibers the enlarged lysosomes in the periphery of the fibers look inconsequential (Raben et al., 2007a).

Autophagy is a dynamic multi-step process which encompasses autophagosome formation, maturation, fusion with lysosomes, and breakdown and recycling of autophagic substrates. The term “autophagic flux” refers to the whole process—the flux is complete if the formation of autophagosomes is followed by their fusion with lysosomes and degradation of the cargo. The failure of a downstream step of autophagy, fusion between autophagosomes and lysosomes, would result in incomplete flux, a condition known as autophagic block (Mizushima et al., 2010). Therefore, the increase in the number of autophagosomes (as shown by a significant increase in the LC3II levels) in Pompe muscle fibers could indicate an upregulation of autophagy or defects in autophagosome-lysosome fusion. An ubiquitin-binding scaffold protein, p62, also known as sequestosome 1 (SQSTM1), is commonly used as a marker of autophagic flux. The protein accumulates in cells from autophagy-deficient mice (Komatsu et al., 2007), and an increase in the level of p62 is an indication of a functional deficiency of autophagy. The protein itself is a substrate for autophagy-mediated degradation; in addition, p62 can directly bind to LC3, thus connecting ubiquitinated (Ub) proteins which are destined for degradation and autophagic machinery (Pankiv et al., 2007; Bjørkøy et al., 2009).

**Figure 3:**
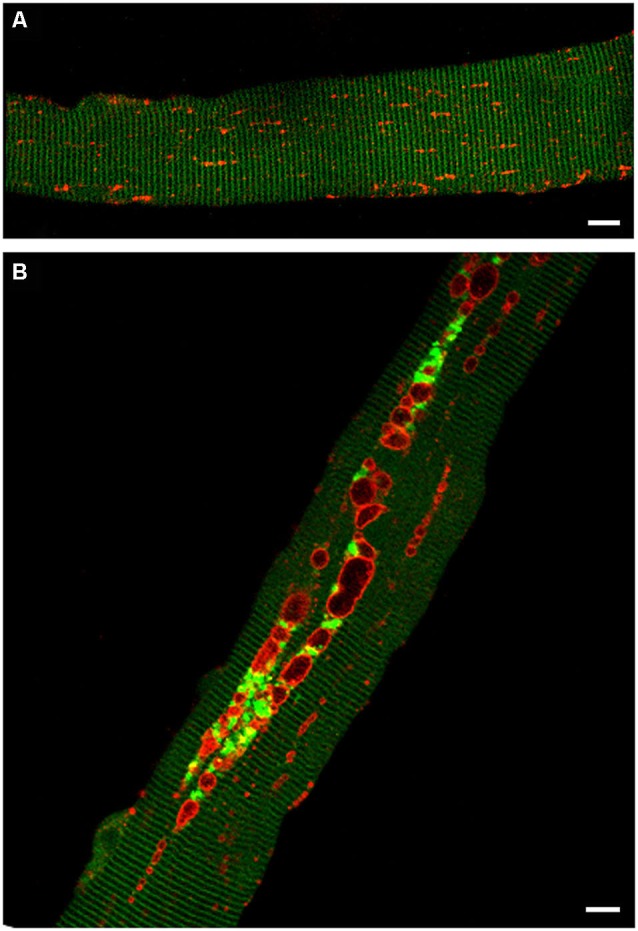
**Confocal microscopy images of fibers from GFP-LC3:WT (A) and GFP-LC3:GAA-KO (B) mice.** In these strains autophagosomes are labeled with green fluorescent protein. Accumulation of autophagosomes (green) can be seen in live unstained muscle fibers derived from GAA-KO, but not from WT mice. Muscle was transfected *in vivo* by electroporation with mCherry-LAMP1 to visualize lysosomes (red); fibers were isolated 6 days after the procedure. **(A)** Autophagosomes are not detectable in the control muscle. **(B)** This still image shows autophagic buildup in the core of a fiber from the diseased muscle. Time-lapse microscopy of mCherry-LAMP1 transfected fibers demonstrated that there is little, if any, fusion between lysosomes and autophagosomes in the areas of autophagic buildup. Bar: 10 μm.

The levels of potentially toxic high molecular mass Ub-proteins in Pompe muscle were significantly increased suggesting a failure of autophagosomal–lysosomal fusion and a block in autophagosomal turnover. The accumulation of Ub-proteins in the GAA-KO muscle, particularly in the non-soluble fraction, preceded the development of clinical symptoms and increased with age. In addition, immunostaining of isolated muscle fibers showed that both the Ub-proteins and p62/SQSTM1 were seen within the autophagic areas, again indicating that the recycling process is inefficient in Pompe skeletal muscle (Raben et al., 2008; Shea and Raben, 2009). A direct evidence of the impaired autophagosomal-lysosomal fusion came from time-lapse microscopy of muscle fibers in which autophagosomes were labeled with GFP-LC3 and lysosomes were labeled with mCherry-LAMP1. These fibers were derived from transgenic GFP-LC3:GAA-KO mice, in which muscles had been *in vivo* transfected with mCherry-LAMP1 (Figure 3). The autophagosomal-lysosomal fusion events were essentially non-existent in the autophagic areas over the course of several hours of time-lapse microscopy (Spampanato et al., 2013).

The autophagic buildup containing cellular debris represents a huge non-contractile inclusion in the diseased muscle fibers. Studies in a different mouse model of Pompe disease, AGLU−/− (Bijvoet et al., 1998), have shown that the enlarged lysosomes in skeletal muscle cannot adequately account for the reduction in mechanical performance, and that the presence of large inclusions greatly contributes to the impairment of muscle function (Hesselink et al., 2003; Drost et al., 2005). Unlike sarcomeres, these inclusions are unable to generate force leading to a significant loss of force per unit of muscle mass. It has also been shown that skeletal muscle weakness in old AGLU−/− mice is caused by both loss of contractility and muscle mass (Hesselink et al., 2003; Drost et al., 2005).

Furthermore, our data demonstrated that the autophagic buildup affects the trafficking and delivery of the recombinant enzyme. The fate of the therapeutic enzyme was analyzed by monitoring the trafficking of the labeled rhGAA (Alexa-Fluor 546) in GFP-LC3:GAA-KO mice. The recombinant enzyme was detected almost exclusively within autophagosomes clustered in the buildup areas (Spampanato et al., 2013). This finding is not unexpected, considering the relationship between the autophagic pathway and the endocytic pathway; as mentioned above, the autophagic and endocytic pathways converge not only at the lysosome, but also at other steps along the way. It appears that the therapeutic drug is diverted away from its intended destination, the lysosome, and instead ends up in the autophagic area, which becomes a sink for the recombinant enzyme (Fukuda et al., 2006; Spampanato et al., 2013). Thus, in Pompe disease, a profoundly disordered intracellular recycling system appears to be an important contributor to the muscle weakness and to the incomplete response to treatment. The autophagic dead-end found in the muscle of Pompe mice is largely limited to muscles rich in glycolytic type II muscle fibers, which are most resistant to therapy, thus confirming the link between the defective autophagy, and the unresponsiveness to ERT with recombinant human enzyme. Abnormal autophagy and toxicity from accumulated Ub material may also be a general mechanism of cellular damage in other lysosomal storage disorders (Lieberman et al., 2012).

### Autophagy in Pompe patients

The apparent disconnect between the findings in Pompe mice, in which dysfunctional autophagy seemed to be a prominent feature of skeletal muscle pathology, and the morphological data (described above) on the stages of the disease progression in infants (Thurberg et al., 2006) raised some skepticism regarding the relevance of mouse studies to human disease. A closer look at muscle biopsies of untreated patients with severe classic infantile form aimed specifically to detect autophagy (namely, immunostaining of isolated fibers for lysosomal and autophagosomal markers) showed that, indeed, the overwhelming characteristic of muscle fibers in infants was the presence of hugely expanded lysosomes often without clear borders, a feature consistent with the long-held hypothesis of lysosomal rupture and the release of glycogen and lytic enzymes into the cytoplasm as a cause of muscle destruction (Griffin, 1984b; Thurberg et al., 2006).

However, analyses of muscle biopsies from patients with milder childhood/juvenile and adult forms of the disease justified the role of autophagy as a critical player in the pathogenesis of Pompe disease in this subset of patients. The pathological changes in many muscle fibers from these patients, even more so than in mice, reflected the autophagic abnormalities leading to muscle destruction. Large autophagic buildup often dwarfs the enlarged glycogen-filled lysosomes that lie outside the autophagic region, and in some fibers the buildup appears to be the only pathology (Raben et al., 2007b; Lewandowska et al., 2008; Raben et al., 2010a); unlike in mice, there is no selective involvement of the different histochemical fiber types. Similar to the findings in the murine model, the defective autophagic flux in muscle of patients with these forms impinges on the maturation of GAA and the uptake of recombinant enzyme during ERT (Nascimbeni et al., 2012a,b).

Notably, autophagic accumulation, closely resembling that in children and adults, becomes prominent (although limited in size) in infants who benefit most from ERT and survive beyond infancy (Raben et al., 2010a). The autophagic buildup in muscle biopsies from these patients is usually seen in fibers with minimal or no lysosomal enlargement outside the autophagic area, suggesting that the therapeutic enzyme reached these lysosomes, and digested the accumulated glycogen. The emergence of autophagic buildup on therapy is clearly not a “side effect” of the treatment, but rather an indication that the drug reverses the pathology only partially, and leaves a subset of lysosomes that are unable to fuse with the autophagosomes. It remains unclear to what extent this autophagic buildup could grow in size in patients on therapy; it is also not clear whether the clinical decline is caused by this emerging pathology.

### Lipofuscinosis

**Figure 4:**
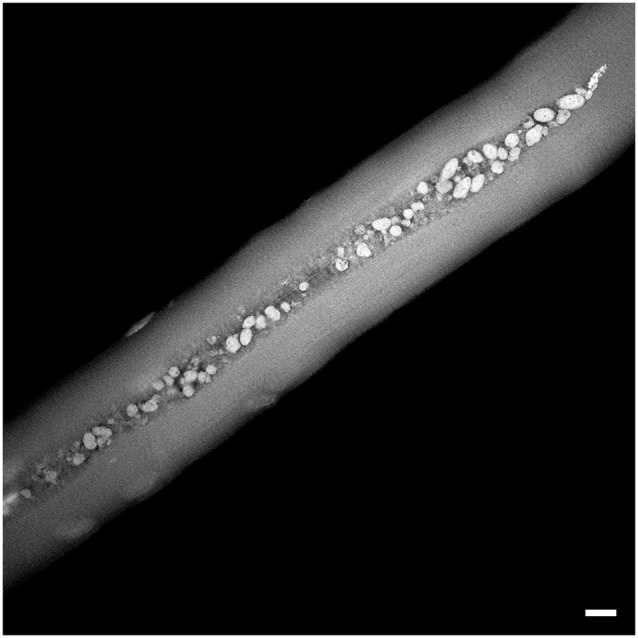
** Autofluorescent lipofuscin inclusions in a muscle biopsy from a patient (NBSL9a) with a childhood form of Pompe disease**. The patient was diagnosed during a family study and began therapy at 7 years of age. The biopsy was taken prior to the initiation of ERT. Bar: 10 μm.

There is yet another abnormality in both untreated and treated children and adults as well as in treated infants which recently came to light: muscle biopsies in the majority of patients contain large, irregularly shaped autofluorescent inclusions (Figure 4). In some patients, more than 75% of fibers contained these structures, which can span up to several hundred microns along the length of the fiber. These structures are usually located in the area of autophagic buildup within LAMP-positive lysosomes or LAMP/LC3-double positive autolysosomes (vesicles formed by autophagosomal-lysosomal fusion) or free in the cytoplasm. Their characteristics—electron density, contrast in transmitted light, a wide-spectrum of autofluorescence, which is quenched by Sudan Black B, and positive staining for lipid markers—unequivocally identify the particles as lipofuscin (Schoser et al., 2007; Feeney et al., 2014). Similar inclusions, although much smaller in size, have been found in muscle of old knockout mice.

Lipofuscin is an autofluorescent lipopigment, which is composed of highly cross-linked undegradable protein aggregates, lipids, carbohydrates, and metals (particularly iron) (Terman et al., 2010). Gradual intralysosomal accumulation of lipofuscin, particularly in terminally differentiated cells (i.e., neurons, retinal pigment epithelium, cardiac myocytes, and muscle cells) is a characteristic sign of cellular oxidative damage and aging (Brunk and Terman, 2002; Terman and Brunk, 2006; Terman et al., 2006). Given the unique role of autophagy and lysosomes in mitochondrial degradation, the diminished degradative capacity of lysosomes due to the progressive deposition of lipofuscin in these organelles would lead to a decrease in the autophagic turn-over of damaged mitochondria, which in turn would result in the generation of reactive oxygen species and formation of oxidized proteins and aggregates, thus perpetuating the production of lipofuscin. In addition, intralysosomal accumulation of lipofuscin affects the trafficking of the newly synthesized lysosomal enzymes, thus further diminishing the degrading capacity of the lysosomes. This “vicious circle” of mitochondrial oxidative damage and lysosomal deposition of lipofuscin is at the heart of a well-known “mitochondrial-lysosomal axis theory of ageing”, which was put forward by Ulf Brunk and Alexei Terman (Brunk and Terman, 2002). According to this model, accumulation of “biological garbage” results from the natural decline in the ability of the cellular degradative machinery to efficiently clear the cells from damaged structures (Terman et al., 2010).

The view of lysosomes as the only site for lipofuscin formation has been recently challenged; it was shown that inhibition of autophagy and reduced lysosomal uptake of protein aggregates did not prevent the formation of lipofuscin, suggesting that lipofuscin can be formed in the cytoplasm (Höhn et al., 2012). If lysosomes are not required for the formation of lipofuscin, then their engulfment by autophagosomes may serve as a protective mechanism designed to mitigate the toxicity (Höhn et al., 2014). In Pompe skeletal muscle, lipofuscin deposits are seen both inside and outside of lysosomes/autophagosomes; the presence of free cytoplasmic lipofuscin may reflect its extralysosomal formation or release into cytoplasm due to lysosomal rupture. Whatever the origin, these deposits are strikingly large in size (Figure 4) and are not associated with advanced age; they are seen in muscle biopsies from patients at different ages, including very young children (Schoser et al., 2007; Raben et al., 2010a; Feeney et al., 2014).

In retrospect, the reported in literature abnormal inclusions in muscle biopsies from Pompe patients—called “reducing body-like inclusions”, “peculiar globular inclusions”, “autofluorescent balloon-like structures”, “acid phosphatase-positive globular inclusions”—all represent lipofuscin (Jay et al., 1992; Sharma et al., 2005; Raben et al., 2010a; Tsuburaya et al., 2012). Given the reduced autophagic activity and a failure of autophagosomal turnover, the presence of lipofuscin in the diseased muscle is not unexpected, but the magnitude of this pathology is. Interestingly, lipofuscin inclusions are often seen in otherwise normal looking fibers. It was suggested that these acid phosphatase-positive/periodic acid Schiff (PAS)-negative inclusions may be a hallmark of the disease and a diagnostic marker, particularly in adult form, which every so often represent the diagnostic challenge (Tsuburaya et al., 2012; Fujimoto et al., 2013).

Thus, our view of the pathogenesis of skeletal muscle damage in Pompe disease (at least in milder childhood and adult forms) evolved from a simple idea of gradual glycogen accumulation and lysosomal expansion to a much more complex picture, which involves a profound dysregulation of autophagy, accumulation of potentially toxic undegradable materials and non-contractile inclusion, as well as oxidative stress. Mitochondrial abnormalities, no doubt, contribute to the pathogenic cascade (our unpublished data). The disease has the characteristics of autophagic myopathy, premature “muscle ageing” or “muscle lipofuscinosis”.

The evolution of our understanding of the pathogenesis of muscle destruction in Pompe disease reflects to a large degree the expansion of the role of the lysosomes themselves. The days of looking at the lysosomes as cellular “garbage disposal unit” or “suicide bags” [the latter term used by Christian de Duve who discovered this organelle (Appelmans et al., 1955)] are long gone. Lysosomes are now viewed more like “the center of cellular universe” (Walkley, 2008). In addition to their role in the digestion and recycling of various extra- and intracellular materials, lysosomes carry multiple tasks—they are implicated in cholesterol homeostasis, plasma membrane repair, tissue remodeling, pathogen defense, MHC class II antigen presentation, down-regulation of surface receptors, and cell death and proliferation (Saftig and Klumperman, 2009). Furthermore, recent studies indicate that lysosomes control their own biogenesis and provide a platform for both activation and deactivation of mTORC1 (mechanistic target of rapamycin complex1), a powerful anabolic regulator of cell growth and metabolism (Zoncu et al., 2011; Bar-Peled and Sabatini, 2014; Demetriades et al., 2014; Menon et al., 2014; Settembre and Ballabio, 2014).

## Experimental approaches to therapy

### Substrate Reduction Therapy (SRT)

Successful application of SRT to other lysosomal diseases (Hollak and Wijburg, 2014) stimulated efforts to test a similar approach in Pompe disease. Inhibition of the two major enzymes involved in glycogen synthesis, glycogenin (GYG) and glycogen synthase (GYS), in primary myoblasts from GAA-KO mice by shRNAs resulted in a decrease in cytoplasmic and lysosomal glycogen accumulation and a reduction in the lysosomal size. A single intramuscular injection of recombinant AAV-1 vectors expressing shGYS into newborn knockout mice led to a significant reduction of glycogen accumulation in skeletal muscle (Douillard-Guilloux et al., 2008). Furthermore, genetic inactivation of GYS1 in the GAA-KO mice reversed cardiac abnormalities, reduced glycogen storage and autophagic buildup, and improved exercise capacity (Douillard-Guilloux et al., 2010). Of note, the disruption of GYS1 (muscle form) in the wild type mice (known as MGSKO) leads to abnormal cardiac development and a severe early perinatal mortality; 90% of GYS1-null pups die due to impaired cardiac function (Pederson et al., 2004). However, the lack of glycogen in skeletal muscle in the surviving 10% mice does not affect either the morphology of this tissues or the animals’ ability to exercise. A severe deficiency of muscle glycogen synthase (mutations in the *GYS1* gene; glycogen storage disease type 0), leading to cardiac arrest, has been described in several families (Kollberg et al., 2007; Cameron et al., 2009; Sukigara et al., 2012), thus highlighting the risk of GYS1 suppression in Pompe disease—only muscle-targeted inhibition of GYS1 can be considered.

### Fiber type conversion

A selective resistance of glycolytic type II muscle fibers (fast muscle) to ERT in the GAA-KO mice combined with massive accumulation of autophagic debris in these fibers suggested that a switch from fast to slow fibers may be beneficial in Pompe disease. A successful fiber type conversion was achieved by transgenic expression of PGC-1α—a transcription factor that promotes mitochondrial biogenesis and muscle remodeling (Lin et al., 2002)—in skeletal muscle of the GAA-KO mice. The conversion of fast muscle into muscle with slow metabolic profile restored autophagic flux, reduced the amount of accumulated Ub-proteins, and completely eliminated autophagic buildup. The absence of buildup was unexpectedly associated with induction rather than suppression of autophagy, as shown by a significant upregulation of several autophagy-related proteins, such as LC3II, GABARAP, Beclin-1, and BNIP3. A concomitant dramatic increase in the number of lysosomes in the PGC-1α-overexpressing muscle accounts for this apparent paradox. However, overexpression of PGC-1α in the diseased muscle failed to improve the ERT, mainly because of the considerable increase in the lysosomal glycogen load (Takikita et al., 2010). Although disappointing, these results are interesting in two respects: (1) a slight PGC-1α-induced increase in the levels of cytoplasmic glycogen (seen in wild type muscle) leads to a massive accumulation of lysosomal glycogen in GAA-deficient muscle, suggesting a high rate of lysosomal glycogen disposal in skeletal muscle. This may explain a much higher requirement for the therapeutic drug in skeletal muscle than in the heart; and (2) the biogenesis of lysosomes and autophagosomes is regulated coordinately.

### Suppression of autophagy in skeletal muscle

The association between autophagic buildup and resistance to therapy in Pompe skeletal muscle suggested that suppression of autophagy would be helpful. Since autophagy is a presumed mechanism of glycogen transport to the lysosomes (Schiaffino and Hanzlikova, 1972; Kotoulas et al., 2006; Schiaffino et al., 2008), this approach also had a potential to reduce or even eliminate lysosomal glycogen accumulation. The genetic suppression of autophagy in GAA-KO mice was achieved by selective inactivation of a critical autophagic gene, Atg7, in skeletal muscles (Atg7/GAA double knockout). Indeed, inactivation of autophagy resulted in a significant decrease in the amount of accumulated glycogen, supporting the idea that autophagic pathway is at least partially responsible for the delivery of glycogen to the lysosomes; microautophagy could be another route by which glycogen enters the lysosome. As expected, autophagic buildup was not observed in the double knockout mice, although small clusters of double-membrane vesicles were detected by electron microscopy. In our experience, the phenotype of these muscle-specific autophagy-deficient GAA-KO mice is not worse (if not better) compared to that of the GAA-KO; however, an assessment of muscle strength by functional tests are needed to clarify this point (Raben et al., 2010b). The loss of autophagy alone in skeletal muscle of wild type mice is associated with the accumulations of dysfunctional mitochondria, mild atrophy, and age-dependent decrease in muscle strength (Masiero et al., 2009; Wu et al., 2009; Masiero and Sandri, 2010). The negative effects of suppression of autophagy in the GAA-KO mice are likely balanced by the beneficial effect of glycogen reduction.

Inactivation of a different autophagic gene, Atg5, in skeletal muscle of the GAA-KO mice, led to a modest glycogen reduction and worsening of clinical manifestations: these Atg5/GAA double knockouts develop early signs of muscle wasting and do not survive beyond 9–12 months of age (both GAA-KO and Atg7/GAA-KO have near normal lifespan) (Raben et al., 2008). The reason for the difference between the two double knockouts (expected to be indistinguishable), is not clear; a various degree of autophagy inactivation due to the difference in the promoters, which were used to excise the autophagic genes (human skeletal actin for the Atg5 and myosin light chain for the Atg7), may explain the discrepancy. However, both autophagy-deficient GAA-KO strains responded remarkably well to ERT—a near complete removal of stored lysosomal glycogen was observed in skeletal muscle—an outcome never seen in the ERT-treated GAA-KO mice. These data established the rational for autophagy-targeted therapy (Raben et al., 2010b).

### Stimulation of lysosomal exocytosis

Perhaps the most intriguing approach is that which exploits the intrinsic property of lysosomes to undergo regulated exocytosis. The process involves translocation and docking of lysosomes to the plasma membrane, followed by fusion with the membrane and release of the lysosomal content into the extracellular space. Initially ascribed to a subset of secretory lysosome-related organelles in hematopoietic cells or melanocytes, this calcium-dependent process is now attributed to all conventional lysosomes—a finding that changed the view of lysosomes as a terminal, dead-end degradation compartment (Andrews, 2000). Furthermore, this ability of lysosomes to exocytose is now seen as an integral part of the lysosomal function—to accomplish cellular clearance by degrading the cargo or by discharging it (Settembre and Ballabio, 2014). However, it was not until the discovery of transcription factor EB (TFEB) that the induction of lysosomal exocytosis became a therapeutic strategy in a variety of lysosomal storage disease and disorders with accumulation of abnormal proteins (Sardiello et al., 2009; Medina et al., 2011; Settembre and Ballabio, 2011).

The bHLH-leucine zipper TFEB, a master regulator of lysosomal biogenesis and autophagy, has been shown to stimulate the generation of new lysosomes and autophagosomes, and to promote fusion of lysosomes with autophagosomes and plasma membrane in a variety of cell types (Sardiello et al., 2009; Settembre and Ballabio, 2011; Settembre et al., 2011). The mechanism of TFEB-mediated gene regulation involves its ability to directly bind a 10-base pair sequence (called CLEAR: Coordinated Lysosomal Expression and Regulation) in the promoter regions of many lysosomal and autophagic genes (Sardiello et al., 2009).

Indeed, overexpression of TFEB in both GAA-KO mice and cultured Pompe muscle cells reduced glycogen load and lysosomal size, improved autophagosome processing, and alleviated excessive accumulation of autophagic vacuoles, thus establishing TFEB as a valid therapeutic target in Pompe disease. Lysosomal docking/fusion with the plasma membrane was visualized during time-lapse microscopy of live muscle fibers isolated from TFEB-treated GAA-KO mice (Feeney et al., 2013; Spampanato et al., 2013). More recent studies showed that a closely related but distinct transcription factor E3 (TFE3) is another target (perhaps even more attractive) since it is abundant in skeletal muscle, whereas TFEB is not. Similar to TFEB, TFE3 binds to the CLEAR elements in the promoter region of multiple lysosomal genes (Martina et al., 2014a,b). Furthermore, mapping TFE3 binding sites across the genome in the wild type muscle cells by ChIP-seq analysis showed similarity to known TFEB binding locations (our unpublished data). Overexpression of TFE3 induced lysosomal exocytosis and promoted glycogen clearance in Pompe muscle cells (Martina et al., 2014b).

The appeal of TFEB or TFE3 modulation as a therapeutic option in Pompe disease is twofold: (1) this approach circumvents the major hurdle of the current therapy—inefficient enzyme delivery to skeletal muscle; and (2) unlike other efforts, it restores autophagic flux and addresses both lysosomal and autophagic pathologies.

## Conclusion and future studies

The limitations of enzyme replacement therapy in Pompe disease have led to reassessment of the basic skeletal muscle pathology, which in turn uncovered new pathogenic mechanisms, in particular the role of autophagy. This new understanding allowed for the identification of novel therapeutic targets that hopefully will guide us toward the development of fresh strategies independent of or complementary to ERT.

In spite of all the advances, many questions still remain. The signaling pathways which are linked to the lysosomes are still an unexplored area in Pompe disease. Recent findings have positioned the lysosome at the center of the mTORC1 signaling pathway; mTORC1 is a protein kinase complex that functions as a central regulator of cellular growth and metabolism by integrating signals from nutrients, energy, and growth factors (Zoncu et al., 2011; Bar-Peled and Sabatini, 2014). A skeleton explanation of the activation and inhibition of the mTORC1 is as follows. Accumulation of amino acids in the lysosomal lumen initiates a signal to a multiprotein lysosome-based complex, which culminates in the mTORC1 recruitment to the lysosome where it is activated by Rheb (Ras homolog enriched in brain). The recruitment and activation of mTORC1 depends on the nucleotide-bound state of the Rags (Bar-Peled and Sabatini, 2014). The inhibition of lysosomal function or depletion of amino acids from the lysosome results in the release of mTORC1 from the lysosome and its inactivation (Zoncu et al., 2011). Perhaps even more relevant to Pompe disease is the data indicating that the Rag family also signals glucose concentration to mTOR; in other words, glucose, like amino acids, controls mTORC1 recruitment to the lysosomal surface and its activation (Efeyan et al., 2013).

Activation of mTORC1 also induces phosphorylation of TFEB and TFE3 and their retention in the cytosol where these transcription factors remain inactive; inhibition of phosphorylation (for example, by nutrient deprivation) stimulates their nuclear translocation and activation leading to the induction of multiple target gens including lysosomal genes (Settembre et al., 2011; Martina et al., 2012; Settembre et al., 2012; Martina et al., 2014b). Thus, the lysosome regulates its own biogenesis by a lysosome-to-nucleus signaling mechanism (Settembre et al., 2012). Therefore, pharmacological inhibition of TFE3 phosphorylation would promote cellular clearance in Pompe disease as well as in other lysosomal storage disorders. The regulation of TFEB and TFE3 in skeletal muscle in general and in Pompe disease in particular remains an open question. This is a rewarding field for future studies.

## Funding

This research was supported in part by the Intramural Research Program of the National Institute of Arthritis and Musculoskeletal and Skin diseases of the National Institutes of Health. Dr. Lim and Dr. Li are supported in part by a CRADA between NIH and Genzyme Corporation and by the Acid Maltase Deficiency Association.

## Conflict of interest statement

The authors declare that the research was conducted in the absence of any commercial or financial relationships that could be construed as a potential conflict of interest.
